# Contribution of Ruminal Fungi, Archaea, Protozoa, and Bacteria to the Methane Suppression Caused by Oilseed Supplemented Diets

**DOI:** 10.3389/fmicb.2017.01864

**Published:** 2017-09-29

**Authors:** Shaopu Wang, Katrin Giller, Michael Kreuzer, Susanne E. Ulbrich, Ueli Braun, Angela Schwarm

**Affiliations:** ^1^Animal Nutrition, Institute of Agricultural Sciences, ETH Zurich, Zurich, Switzerland; ^2^Animal Physiology, Institute of Agricultural Sciences, ETH Zurich, Zurich, Switzerland; ^3^Clinic for Ruminants, Vetsuisse Faculty, University of Zurich, Zurich, Switzerland

**Keywords:** rumen, ruminant, rumen microorganisms, methanogenesis, camelina, safflower, poppy

## Abstract

Dietary lipids can suppress methane emission from ruminants, but effects are variable. Especially the role of bacteria, archaea, fungi and protozoa in mediating the lipid effects is unclear. In the present *in vitro* study, archaea, fungi and protozoa were selectively inhibited by specific agents. This was fully or almost fully successful for fungi and protozoa as well as archaeal activity as determined by the methyl-coenzyme M reductase alpha subunit gene. Five different microbial treatments were generated: rumen fluid being intact (I), without archaea (–A), without fungi (–F), without protozoa (–P) and with bacteria only (–AFP). A forage-concentrate diet given alone or supplemented with crushed full-fat oilseeds of either safflower (*Carthamus tinctorius*) or poppy (*Papaver somniferum*) or camelina (*Camelina sativa*) at 70 g oil kg^−1^ diet dry matter was incubated. This added up to 20 treatments with six incubation runs per treatment. All oilseeds suppressed methane emission compared to the non-supplemented control. Compared to the non-supplemented control, –F decreased organic matter (OM) degradation, and short-chain fatty acid concentration was greater with camelina and safflower seeds. Methane suppression per OM digested in –F was greater with camelina seeds (−12 vs.−7% with I, *P* = 0.06), but smaller with poppy seeds (−4 vs. −8% with I, *P* = 0.03), and not affected with safflower seeds. With –P, camelina seeds decreased the acetate-to-propionate ratio and enhanced the methane suppression per gram dry matter (18 vs. 10% with I, *P* = 0.08). Hydrogen recovery was improved with –P in any oilseeds compared to non-supplemented control. No methane emission was detected with the –A and –AFP treatments. In conclusion, concerning methanogenesis, camelina seeds seem to exert effects only on archaea and bacteria. By contrast, with safflower and poppy seeds methane was obviously reduced mainly through the interaction with protozoa or archaea associated with protozoa. This demonstrated that the microbial groups differ in their contribution to the methane suppressing effect dependent on the source of lipid. These findings help to understand how lipid supplementation and microbial groups interact, and thus may assist in making this methane mitigation tool more efficient, but await confirmation *in vivo*.

## Introduction

The main microbial groups inhabiting the rumen are anaerobic bacteria, archaea, fungi and protozoa, which contribute directly or indirectly to dietary organic matter (OM) degradation. The bacteria are most abundant with an estimated population density of 10^10−11^ mL^−1^ of rumen fluid, followed by archaea (10^8−9^ mL^−1^; all of them methanogens), ciliate protozoa (10^6^ mL^−1^) which contribute up to half of the rumen microbial biomass due to their large size, and fungi with 10^6^ mL^−1^ contributing less than 8% to total biomass (Orpin and Joblin, 1997; Lourenço et al., 2010; Kumar et al., 2013). A broad knowledge on the capacities and actual contribution of these microbial groups within the rumen has been generated (e.g., Hobson and Stewart, 2012). Methane (CH_4_), one of the natural by-products of ruminal fermentation, is mainly generated by the archaea by utilizing carbon dioxide (CO_2_) and hydrogen (H_2_), both originating mostly from the fiber degrading activity of bacteria, protozoa and fungi. Methane formation contributes to the proper function of the rumen, as it counteracts the inhibition of other microorganisms caused by the accumulation of H_2_, thereby allows more feed being fermented in the rumen (Kumar et al., 2014). The capturing of H_2_ generated by another microbial species is referred to as interspecies H_2_ transfer (Morgavi et al., 2010). This happens between archaea and the other microbial groups. In this respect, the role of the ruminal fungi and their functional importance for CH_4_ formation is still not well known.

Due to the great global warming potential of CH_4_, mitigation strategies through dietary changes, animal management and genetic selection are currently in the focus of scientific and public interest (Cottle et al., 2011; Hristov et al., 2013). Among the nutritional strategies, dietary lipids and feeds rich in lipids have evolved to be among the most promising measures (e.g., reviews by Beauchemin et al., 2008; Hristov et al., 2013). Despite intensive research, the understanding of the mode of action of lipids on ruminal microorganisms and finally on CH_4_ production is limited. Dependent on the type of lipid, certain rumen microbial species are inhibited, while others remain unaffected. The effect may result from a direct toxicity against protozoa and archaea (Machmüller et al., 2003; Zhou et al., 2015). In addition, lipids may have toxic effects on cellulolytic bacteria (Newbold et al., 2015). There are also indirect lipid influences like making nutrients (fiber) inaccessible to the microorganisms through coating. In addition, the biohydrogenation of polyunsaturated fatty acids (PUFA) could act as an alternative H_2_ sink, thus selectively reducing the substrate for the archaea. However, only about 1–2% of the total H_2_ available is utilized by this pathway (Czerkawski and Clapperton, 1984; Hristov et al., 2013). The effectiveness of mitigating CH_4_ by lipids may be related to their fatty acid composition (recently described by Wang et al., 2017). Despite these findings, the shifts occurring in, and the quantitative role of, the individual main microbial groups in this respect are not well known. The main reason for that is the difficulty to separate the contribution of the individual groups in the complex rumen ecosystem.

In the present study, we hypothesized that the different groups of ruminal microorganisms may have a different quantitative importance for the suppression of CH_4_ formation caused by lipids. For this purpose, we modified rumen fluid in a way that either only one of the four distinct groups was inhibited or all groups except bacteria. In order to investigate the role of the type of lipid, three oilseeds effective in CH_4_ mitigation but largely differing in their composition of PUFA (Wang et al., 2017) were chosen as test lipids. This should foster the exhibition of interactions between lipid type and microbial group.

## Materials and methods

### *In vitro* technique

The Hohenheim gas test apparatus was employed, and the setup and operation were as described in the protocol of Soliva and Hess (2007). Syringes with two outlets, one for filling and decanting the liquid and one covered with a polyfluoroethylene-layer for gas sampling, were used. The donor of the rumen fluid was a lactating rumen-cannulated Brown Swiss cow. The experimental protocol complied with the Swiss legislation for Animal Welfare and was approved by the Committee on Animal Experimentation of the Cantonal Veterinary Office Zurich (approval number ZH 38/14). The cow was fed *ad libitum* on grass hay and silage (66:33) and received 0.5 kg day^−1^ of concentrate (UFA 142, UFA AG, Herzogenbuchsee, Switzerland) and minerals (UFA 195). The rumen fluid was collected before morning feeding, stored in a pre-warmed thermos bottle and was processed in the laboratory within 1 h. Prior to use, the rumen fluid was strained through four layers of gauze (1 mm pore size) in order to remove the solid particles. Afterwards it was added to a pre-warmed buffer (39°C) in a ratio of 33:66 (rumen fluid to buffer). Under a continuous stream of CO_2_ to maintain anaerobic conditions, 30 mL of the buffered rumen fluid was dispensed into each syringe pre-filled with 200 mg dry matter (DM) of a basal diet consisting of hay and concentrate with a ratio of 60:40 in DM (ether extract, neutral detergent fiber and crude protein, 35, 404, and 165 g kg^−1^ DM). Oilseeds were added in the main experiment on top of the basal diet. In each run, additionally to the experimental treatments, three syringes were incubated without feed to serve as blanks for adjusting treatment data to net gas production. After incubation for 24 h at 39°C, the gas volume was read from the calibrated scale on each glass syringe. The gas from fermentation was kept in the syringes for analysis of contents of CH_4_ and gaseous hydrogen (gH_2_).

### Generation of the experimental microbial treatments

For the selective inhibition of archaea, fungi and protozoa, 20 mmol L^−1^ of bromoethanesulfonate (BES) (Poulsen et al., 2013), 0.5 mg of cycloheximide mL^−1^ (Dehority and Tirabasso, 2000) and 2.5 mL of synperonic NP9 L^−1^ (Dohme et al., 1999), respectively, were added to the buffered rumen fluid. A final treatment was a manipulation with only bacterial activity left. For this purpose, all three above mentioned inhibitors were added altogether to the buffered rumen fluid. This yielded five different buffered rumen fluid types, one remaining intact (I), one without archaeal activity (–A), one without fungal activity (–F), one without protozoal activity (–P) and one with only bacterial activity left (–AFP).

### Preliminary experiment with different microbial groups

In order to assess the time required to achieve a substantial inhibition of the target microbial groups by the inhibitors, the formation of CH_4_, gH_2_ and CO_2_ was monitored after 1, 2, 4, 6, 12, and 24 h of incubation. For this purpose, the five microbial treatments were incubated with the basal diet. Total gas production was recorded and gas samples from each treatment were collected from different syringes reserved for each of the time points. Aliquots of 0.15 mL fermentation gas were withdrawn from each syringe using a gas-tight Hamilton syringe. The gas samples were immediately analyzed for their composition. Four runs were conducted at four different days resulting in *n* = 4 per microbial treatment and time point.

### Main experiment

Microbial treatments were generated as described above. Based on the results of the preliminary experiment, where plateaus in CH_4_ and gH_2_ concentrations were mostly established during the first 2 h, this time of pre-incubation of the buffered rumen fluid with the inhibitors but without diet was applied. The pre-incubation took place under continuous flow of CO_2_ to maintain anaerobic conditions at 39°C. These 2 h of pre-incubation were followed by 24 h incubation with the diets at 39°C. The microbial treatments were tested either without or with one of three oilseeds. Seeds of safflower (*Carthamus tinctorius*), poppy (*Papaver somniferum*), and camelina (*Camelina sativa*) were purchased from Exoticsamen (Sulzbach-Rosenberg, Germany), Alfred Galke (Bad Grund, Germany) and Zollinger Samen (Les Evouettes, Switzerland), respectively. In the case of safflower, a mixture containing equal proportions in DM of kernels with and without husk were used in order to minimize the difference in the nutrient composition of the three oilseeds contrasting in their fatty acid profile. The detailed fatty acid profile and nutrient composition of the same batches as those used in the present study can be found in Wang et al. (2017). The seeds of safflower were characterized by high proportions of linoleic acid (two double bonds) and low proportions of α-linolenic acid (three double bonds) with 810 and 1 g kg^−1^ total fatty acids, respectively. The corresponding proportions (g kg^−1^ total fatty acids) in the seeds of camelina were opposite with 162 and 358 and those of the seeds of poppy were in between with 720 and 6. In Wang et al. (2017) we showed that these seeds differ in their efficiency in mitigating ruminal methane formation. All oilseeds were ground through a 1-mm screen with a centrifugal mill before incubation. Supplementations comprised 70 g seed lipids (ether extract) kg^−1^ basal diet DM. This meant DM additions per syringe of 35, 28.5, and 39.4 mg of safflower, poppy, and camelina seeds, respectively. After terminating incubations, the liquid in the syringes was decanted into tubes for immediate pH and ammonia measurements. Besides, aliquots of 1 mL liquid were collected in 1.5 mL Eppendorf tubes, frozen in liquid nitrogen and then stored at −80°C for microbial analysis. For quantification of total short-chain fatty acid (SCFA), the incubation fluid was centrifuged at 4,000 g for 5 min at 4°C and stored frozen at −20°C until analysis. Aliquots of 0.15 mL fermentation gas were withdrawn from each syringe using a gas-tight Hamilton syringe. Six separate runs were carried out on six different days distributed across 1.5 months where each treatment was always represented in duplicate. These duplicate values were averaged for statistical evaluation resulting in *n* = 6 for each of the 20 experimental treatments. For SCFA 5 replicates, not 6 were available.

### Laboratory analysis

The pH and ammonia concentration were measured in the incubation fluid using a digital pH meter (Metrohm model 632 and 713, respectively, Herisau, Switzerland) connected with the glass electrodes 6.0204.100 and electrode 6.0506.100 (Metrohm), respectively. The fermentation gas samples were analyzed for the concentrations of CH_4_, gH_2_, and CO_2_ (preliminary experiment only) with a gas chromatograph (6890N, Agilent Technologies, Wilmington, DE, USA) equipped with a thermal conductivity detector. Total SCFA and their proportions were analyzed by HPLC (LaChrom, L-7000 series; Hitachi Ltd, Tokyo, Japan) equipped with a UV detector by the method of Ehrlich et al. (1981). Analysis of DM and crude ash content in the feeds were conducted using an automated thermogravimetric analyzer (TGA 701, Leco Corporation, St Joseph, Michigan, USA; AOAC, 1997 index no. 942.05).

Total RNA was extracted from the incubation fluid samples using Trizol (TRI Reagent, Sigma, St. Louis, USA) combined with mechanical disruption by bead beating. After thawing the samples on ice, a volume of 0.5 mL incubation fluid was mixed with 1 mL Trizol in tubes pre-filled with 1.0 mm silica glass beads (BeadBug™ prefilled tubes, 2.0 mL capacity, Sigma). The mixed samples were disrupted in the MagNA Lyser (Roche Diagnostics, Rotkreuz, Switzerland) three times at 7,000 rpm for 45 s, and cooled down in the cooling block between mixing. The disrupted samples were transferred into new 2.0 mL tubes, 300 μL chloroform was added to the samples and they were vortexed for 15 s. After incubating the samples at room temperature (RT) for 3 min, they were centrifuged at 12,000 g for 15 min at 4°C. The supernatant containing the RNA was mixed with 500 μL isopropanol in a new tube, vortexed for 15 s and incubated at RT for 10 min before being centrifuged at 12,000 g for 15 min at 4°C. The supernatant was discarded and 1 mL of 75% ethanol was added to wash the RNA pellets. After vortexing and centrifugation at 7,500 g for 5 min at 4°C, the supernatant was removed and the RNA pellets were dried at RT. The RNA was dissolved in 20 μL nuclease-free water and frozen at −80°C for later analysis.

In order to remove potential contamination caused by genomic DNA, the extracted total RNA was purified by the RNase-Free DNase Set kit prior to RNA cleanup and concentration with the RNeasy MinElute Cleanup Kit following the protocol of Qiagen AG (Hombrechtikon, Switzerland). The concentrations of extracted and purified RNA were quantified using the NanoDrop 2000 (peqLab, Erlangen, Germany). The integrity of the RNA was assessed by a Bioanalyzer 2100 (Agilent Technologies, Waldbronn, Germany) and the Agilent RNA 6000 Nano Kit. The RNA integrity numbers were between 6 and 10 for all samples. The RNA concentration of each sample was adjusted to 500 ng in a volume of 10 μL, which was reversely transcribed with GoScript™ Reverse Transcription System (Promega AG, Dübendorf, Switzerland) with the following reaction mix: 0.5 mL Oligo(dT)_15_ primer, 0.5 μL random primer, 4.0 μL reaction buffer, 2.5 μL MgCl_2_, 1 μL dNTPs, 0.5 μL RNasin, 1 μL reverse transcriptase. The reaction mix was incubated in a PCR cycler with the following conditions: 5 min at 25°C, 60 min at 42°C and 15 min at 70°C. Quantitative real-time PCR (qPCR) was performed on a CFX384 Real-Time PCR Detection System (Bio-Rad, Munich, Germany) with the KAPA SYBR FAST qPCR Kit (Kapa Biosystems, Wilmington, MA, USA) with the following cycle conditions: initial step of 30 s at 95°C and 40 cycles of 3 s at 95°C and 20 s at 60°C followed by a dissociation curve analysis. The reaction volume was 10 μL consisting of 5 μL Master Mix, 0.4 μL of each primer (10 μM), 0.07 μL VisiBlue (TATAA Biocenter, Göteborg, Sweden), 3.13 μL water, and 1 μL cDNA. The primer sequences used are listed in Table 1. In this respect, the methyl-coenzyme M reductase alpha subunit gene (*mcrA*) is coding for the enzyme of the terminal step of the methanogenesis pathway (Guo et al., 2008) and thus is an indicator of the activity of the archaea.

**Table 1:** Primer sets used in the real time PCR analysis of ruminal microbiota^a^.

**Target taxon**	**Primer set**	**Primer sequence 5′–3′**	**References**
Total archaea	Arch f	GYGCAGCAGGCGCGAAA	Zeitz et al., 2012
	Arch r	GGACTACCSGGGTATCTAAT	
Total fungi	Denfun f	GAGGAAGTAAAAGTCGTAACAAGGTTTC	Denman and McSweeney, 2006
	Denfun r	CAAATTCACAAAGGGTAGGATGATT	
Total protozoa	PSSU-316f	GCTTTCGWTGGTAGTGTATT	Sylvester et al., 2004
	PSSU-316f	CTTGCCCTCYAATCGTWCT	
Total bacteria	Denbac f	CGGCAACGAGCGCAACCC	Denman and McSweeney, 2006
	Denbac r	CCATTGTAGCACGTGTGTAGCC	
*mcrA* gene^b^	qmcrA F	TTCGGTGGATCDCARAGRGC	Denman et al., 2007
	qmcra R	GBARGTCGWAWCCGTAGAATCC	
*Fibrobacter*	Fisu f	GGRCGGGATTGAATGTAC	Zeitz et al., 2012
*succinogenes*	Fisu r	AATCCGCTTGAATCTCCG	
*Ruminococcus*	Ra1281 f	CCCTAAAAGCAGTCTTAGTTCG	Koike and Kobayashi, 2001
*albus*	Ra1439 r	CCTCCTTGCGGTTAGAACA	
*Ruminococcus*	Rflav f	TGTCCCAGTTCAGATTGCAG	Zeitz et al., 2012
*flavefaciens*	Rflav r	GGCGTCCTCATTGCTGTTAG	

a *qPCR was run at an annealing temperature of 60°C for all primers*.

b *mcrA gene: methyl-coenzyme M reductase alpha subunit gene*.

### Calculations and statistical analysis

The gas production in the blank was subtracted from the gas production recorded from the syringes incubated with feed to obtain the net amount of gas produced. The *in vitro* OM digestibility (IVOMD) was calculated using the standard equation of Menke and Steingass (1988) as IVOMD (g kg^−1^) = 148.8 + 8.893 × total gas production (mL 200 mg^−1^ DM) + 0.448 × crude protein (CP, g kg^−1^ DM) + 0.651 × ash (g kg^−1^ DM). The amount of OM digested was calculated from OM supply and IVOMD. The H_2_ balance, comprising H_2_ generated, H_2_ utilized and H_2_ recovery, was calculated by considering SCFA and CH_4_ formation during *in vitro* fermentation (Demeyer, 1991). The respective equations were as follows:

(1) $$\begin{array}{l} {\text{H}}_{2}\text{ generated }(\text{mmol})=\\ \;2\;\times \text{ acetate }(\text{mmol})+1\;\times \text{ propionate }(\text{mmol})\\ \;+\;4\;\times \text{ butyrate }(\text{mmol})+2\;\times \text{ valerate }(\text{mmol})\\ \;+\;2\;\times \text{ isovalerate }(\text{mmol}) \end{array}$$

(2) $$\begin{array}{l} {\text{H}}_{2}\text{ utilized }(\text{mmol})=\\ \;2\;\times \text{ propionate }(\text{mmol})+2\;\times \text{ butyrate }(\text{mmol})\\ \;+\;1\;\times \text{ valerate }(\text{mmol})+4\;\times \;{\text{CH}}_{4}\;(\text{mmol}) \end{array}$$

(3) $$\begin{array}{l} {\text{H}}_{2}\text{ recovery}(\%)=\\ \;100\;\times \;{\text{H}}_{2}\text{ utilized }(\text{mmol})/{\text{H}}_{2}\text{ generated }(\text{mmol}) \end{array}$$

The dissolved H_2_ was estimated using the formula of Wiesenburg and Guinasso (1979) which was applied for rumen fluid by Wang et al. (2016a) a as follows:

$$\begin{array}{l} {\text{ eGas}}_{\left(\text{aq}\right)}={\text{Gas}}_{\left(\text{g}\right)}\alpha_{\text{H2}}\text{Pt}\\ \text{with}\alpha_{\text{H2}}=\exp(-47.8948+65.0368(100/\text{T})\\ \;+\;20.1709\text{ln}(\text{T}/100)) \end{array}$$

where eGas_(aq)_ is the estimated concentration of the dissolved hydrogen (μM), Gas_(g)_ is the corresponding gas concentration measured in the headspace (μM), α_H2_ is the Bunsen absorption coefficient for hydrogen at 1 atm pressure calculated as a function of absolute temperature (T, degrees Kelvin) for a room temperature of 20°C and Pt is 0.95 atm when hydrogen was measured.

Total archaea, fungi, protozoa and bacteria, as well as the archaeal *mcrA* gene and the bacterial species *Fibrobacter succinogenes, Ruminococcus albus* and *Ruminococcus flavefaciens*, were expressed as a proportion of the respective microbial group or species or gene in the I treatment. The ΔCt values per target gene were calculated by subtracting Ct values of the I treatment from the Ct values of the treatments –A, –F, –P, and –AFP. The relative expression of different groups was calculated from the ΔCt values as 2^−ΔCt^. The changes in the microbial groups, the archaeal gene expression and the individual bacterial species in response to the supplementation of the inhibitors were determined by setting their respective RNA expression in the I treatment as 100%.

Data of the main experiment were subjected to analysis of variance using the Mixed procedure of SAS (version 9.3, SAS Institute, Cary, NC). The model applied for the entire dataset included microbial treatment, dietary treatment and the interaction as the fixed effects, and run as random effect. In addition, the effects of the microbial treatments within the individual dietary treatments and the effects of the dietary treatments within individual microbial treatments were analyzed using a single-fixed effect analysis and considering run as random effect. All multiple comparisons among means were performed with Tukey's method. The significance of the differences in the respective microbial group or species or gene or the suppression extent of CH_4_ caused by specific oilseeds between modified treatments and I treatment was analyzed by Student's paired *t*-test with SAS. A level of *P* < 0.05 was considered significant and *P* < 0.10 as a trend.

## Results

### Evolution of the changes in fermentation gases caused by the microbial inhibitors

**Figure 1:**
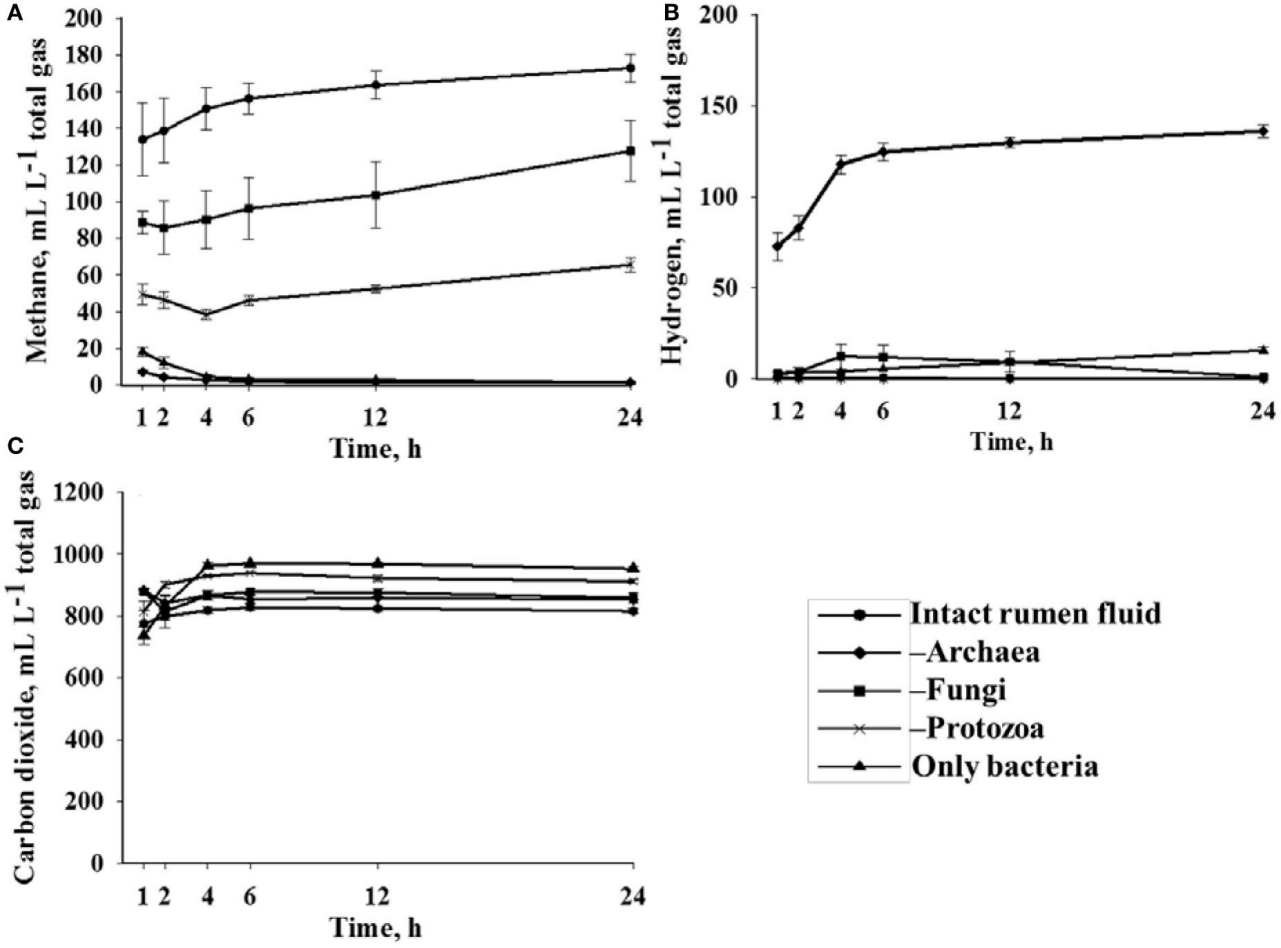
Concentration of **(A)** methane, **(B)** hydrogen, and **(C)** carbon dioxide (mL L^−1^ total fermentation gas) from incubation with the basal diet *in vitro* as determined with Intact rumen fluid and in the absence of different microbial groups (preliminary experiment). *n* = 4 (*n* = 3 for “Only bacteria” at 6, 12, and 24 h), mean ± standard error.

In the preliminary experiment, the inhibitors caused substantial differences in the formation of CH_4_ (decline down to zero; Figure 1A) and gH_2_ (increase; Figure 1B), but not in CO_2_ production (minor decline, Figure 1C). Substantial differences in the production of CH_4_ and gH_2_ occurred already within the first 2 h after exposure to the different inhibitors.

### Effects of the inhibitor treatments on RNA abundance of microbial groups

In the main experiment, inhibitor treatments –A and –AFP reduced (*P* < 0.05) the RNA abundance of the total archaea and the archaeal *mcrA* gene, measured after 26 h (2 h plus 24 h) of incubation (Table 2). Compared to the intact rumen fluid, treatments –F and –AFP reduced (*P* < 0.05) the RNA abundance of the fungi, and –P was free (*P* < 0.05) and –AFP was almost free (*P* < 0.05) from protozoal RNA. Total bacterial RNA abundance increased with the inhibitor treatments –A and –AFP (*P* < 0.10) and –P (*P* < 0.05), and no difference was found with treatment –F. This was different for the abundance of the RNA of the three bacterial species (*F. succinogenes, R. albus* and *R. flavefaciens*), where a decline (*P* < 0.05) almost to zero was observed with the antiprotozoal treatment (–P and –AFP), no difference with –A and an increase (*P* < 0.10) in *R. flavefaciens* with –F. Treatment –P also adversely affected (*P* < 0.05) the RNA abundance of total archaea, archaeal *mcrA* gene and total fungi.

**Table 2:** Relative changes *in vitro* in ruminal microbial groups, archaeal *mcrA* gene expression and selected bacterial species (% of those found in intact rumen fluid) as caused by inhibitor application measured after 24 h incubation with the basal diet without seed supplements (main experiment).

**Microorganisms (groups)**	**Treatment**				
	**–Archaea**	**–Fungi**	**–Protozoa**	**Only bacteria**	**SEM**
Total archaea	32.6^*^	181	27.4^*^	16.9^*^	19.7
Total fungi	82.3	1.7^*^	8.1^*^	2.6^*^	14.6
Total protozoa	52.1	62.0	0.0^*^	0.3^*^	12.3
Total bacteria	172^#^	331	588^*^	316^#^	51.9
*mcrA* gene	3.6^*^	131	4.6^*^	2.0^*^	16.8
*F. succinogenes*	419	235	0.0^*^	0.1^*^	48.5
*R. albus*	342	546	0.0^*^	0.0^*^	69.6
*R. flavefaciens*	95.9	266^#^	0.4^*^	0.6^*^	27.7

mcrA gene, methyl-coenzyme M reductase alpha subunit gene. Difference compared with intact rumen fluid:

* P < 0.05;

# *P < 0.10*.

### General effects of the dietary oilseeds on fermentation and methane emission

When incubated with the intact rumen fluid, the oilseed supplements increased (*P* < 0.05) ammonia concentration (except for poppy) and the amount of OM digested but decreased IVOMD; pH was unaffected (Table 3). The oilseeds had no effect on total SCFA and the proportions of the major SCFA, but camelina enhanced (*P* < 0.05) proportions of valerate, iso-valerate and tended (*P* < 0.10) to increase iso-butyrate; safflower increased (*P* < 0.05) valerate proportions (Tables 4, 5). All seeds decreased (*P* < 0.05) CH_4_ formation per unit of DM or OM digested, but not per unit of SCFA generated. The gH_2_ formation per unit of DM was not affected by the oilseeds (Table 6). Supplementing camelina enhanced (*P* < 0.05) the calculated amount of total H_2_ utilized, otherwise there were no effects on H_2_ balance and dissolved H_2_ (Table 7).

**Table 3:** Effects of the inhibition of different rumen microbial groups and of the supplementation of different oilseeds on pH, ammonia concentration and organic matter degradation.

**Traits/oilseeds**	**Treatment**					**SEM**	***P*****-values**		
	**Intact rumen fluid**	**–Archaea**	**–Fungi**	**–Protozoa**	**Only bacteria**		**Treatment**	**Oilseed**	**Interaction**
**pH**						0.006	<0.001	<0.001	0.011
Control	6.86^x^	6.80^y^	6.83^a^^y^	6.91^a^^w^	6.91^a^^w^		<0.001		
Safflower	6.86^w^	6.80^x^	6.79^b^^x^	6.86^b^^w^	6.87^b^^w^		<0.001		
Poppy	6.85^w^^x^	6.81^y^	6.82^a^^b^^x^^y^	6.86^b^^w^	6.86^b^^c^^w^		<0.001		
Camelina	6.83^w^^x^	6.79^y^	6.81^a^^b^^x^^y^	6.84^b^^w^	6.84^c^^w^^x^		<0.001		
Mean	6.85^w^	6.80^x^	6.81^x^	6.87^w^	6.87^w^		<0.001		
*P*-values	0.14	0.22	0.046	<0.001	<0.001				
**Ammonia (mmol L**^−1^**)**						0.19	<0.001	<0.001	0.019
Control	13.5^b^^w^	12.3^b^^x^	12.4^b^^x^	12.2^b^^x^	12.0^(b)^^x^		0.007		
Safflower	15.4^a^^w^	13.9^a^^x^^y^	14.3^a^^w^^x^	13.0^a^^b^^y^^z^	12.4^(ab)^^z^		<0.001		
Poppy	13.4^b^^w^	12.9^b^^w^^x^	12.8^b^^w^^x^	12.4^a^^b^^w^^x^	12.3^(ab)^^x^		0.027		
Camelina	15.5^a^^w^	14.6^a^^w^^x^	14.3^a^^x^	13.2^a^^y^	13.0^(a)^^y^		<0.001		
Mean	14.5^w^	13.4^x^	13.4^x^	12.7^x^^y^	12.4^y^		<0.001		
*P*-values	<0.001	<0.001	<0.001	0.020	0.050				
**Organic matter digested (mg)**						1.68	<0.001	<0.001	0.69
Control	131^c^^w^	133^b^^w^	115^b^^x^	98^b^^y^	95^c^^y^		<0.001		
Safflower	145^b^^w^	148^a^^w^	129^a^^x^	111^a^^y^	108^a^^b^^y^		<0.001		
Poppy	144^b^^w^	146^a^^w^	131^a^^x^	109^a^^y^	107^b^^y^		<0.001		
Camelina	152^a^^w^	150^a^^w^	134^a^^x^	112^a^^y^	112^a^^y^		<0.001		
Mean	143^w^	144^w^	127^x^	108^y^	106^y^		<0.001		
*P*-values	<0.001	<0.001	<0.001	<0.001	<0.001				
***In vitro*** **organic matter digestibility (g kg**^−1^**)**						7.6	<0.001	<0.001	0.50
Control	707^a^^w^	715^a^^w^	621^x^	529^a^^y^	513^(a)^^y^		<0.001		
Safflower	660^c^^w^	672^b^^w^	586^x^	505^b^^c^^y^	491^(b)^^y^		<0.001		
Poppy	676^b^^c^^w^	686^b^^w^	618^x^	516^a^^b^^y^	505^(ab)^^y^		<0.001		
Camelina	682^b^^w^	673^b^^w^	600^x^	502^c^^y^	503^(ab)^^y^		<0.001		
Mean	681^w^	687^w^	606^x^	513^y^	503^y^		<0.001		
*P*-values	<0.001	<0.001	0.10	<0.001	0.080				

a–c *Within a column, means without a common superscript differ, P < 0.05; superscripts in brackets indicate a trend of a difference among means, P < 0.10*.

w–z *Within a row, means without a common superscript differ, P < 0.05*.

*SEM, the overall standard error of the mean*.

**Table 4:** Effects of the inhibition of different rumen microbial groups and of the supplementation of different oilseeds on total short-chain, C_2_ and C_3_ fatty acids.

**Traits/oilseeds**	**Treatment**					**SEM**	***P*****-values**		
	**Intact rumen fluid**	**–Archaea**	**–Fungi**	**–Protozoa**	**Only bacteria**		**Treatment**	**Oilseed**	**Interaction**
**Total short-chain fatty acid (mmol L**^−1^ **rumen fluid)**						1.20	<0.001	<0.001	0.61
Control	87.4^v^	83.0^c^^w^	85.0^c^^v^^w^	76.5^b^^x^	74.6^c^^x^		<0.001		
Safflower	90.9^v^	86.1^a^^b^^w^	89.0^a^^b^^v^^w^	77.5^b^^x^	77.1^a^^b^^x^		<0.001		
Poppy	92.7^v^	84.1^b^^c^^w^^x^	87.6^b^^c^^v^^w^	77.0^b^^x^^y^	75.0^b^^c^^y^		<0.001		
Camelina	95.2^v^	88.1^a^^w^	90.8^a^^w^	79.8^a^^x^	77.7^a^^x^		<0.001		
Mean	91.5^v^	85.3^w^	88.1^w^	77.7^x^	76.1^x^		<0.001		
*P*-values	0.12	<0.001	<0.001	<0.001	0.005				
**Acetate (mmol mol**^−1^**)**						4.7	<0.001	<0.001	0.76
Control	683^v^	626^a^^b^^x^	657^a^^b^^w^	588^a^^y^	569^(a)^^z^		<0.001		
Safflower	674^v^	628^a^^w^	659^a^^v^	581^a^^b^^x^	567^(ab)^^x^		<0.001		
Poppy	683^v^	632^a^^w^	661^a^^v^	578^a^^b^^x^	563^(ab)^^x^		<0.001		
Camelina	671^v^	619^b^^x^	650^b^^w^	576^b^^y^	553^(b)^^z^		<0.001		
Mean	678^v^	626^x^	657^w^	581^y^	563^z^		<0.001		
*P*-values	0.32	0.004	0.005	0.026	0.066				
**Propionate (mmol mol**^−1^**)**						6.8	<0.001	0.011	0.89
Control	172^x^	213^(b)^^w^	203^(ab)^^w^	316^(b)^^v^	331^b^^v^		<0.001		
Safflower	177^x^	216^(ab)^^w^	202^(b)^^w^	324^(x)^^v^	334^a^^b^^v^		<0.001		
Poppy	174^x^	217^(ab)^^w^	204^(ab)^^w^	325^(a)^^v^	336^a^^b^^v^		<0.001		
Camelina	178^z^	219^(a)^^x^	204^(a)^^y^	326^(a)^^v^	345^a^^v^		<0.001		
Mean	175^z^	216^x^	203^y^	323^w^	336^v^		<0.001		
*P*-values	0.60	0.074	0.095	0.051	0.038				
**Acetate-to-propionate ratio (x:1)**						0.088	<0.001	0.067	0.98
Control	3.99^v^	2.95^a^^x^	3.25^a^^b^^w^	1.87^a^^y^	1.72^(a)^^y^		<0.001		
Safflower	3.82^v^	2.92^a^^b^^x^	3.28^a^^w^	1.80^a^^b^^y^	1.71^(ab)^^y^		<0.001		
Poppy	3.97^v^	2.92^a^^b^^w^	3.26^a^^b^^w^	1.79^a^^b^^x^	1.68^(ab)^^x^		<0.001		
Camelina	3.78^v^	2.83^b^^x^	3.20^b^^w^	1.77^b^^y^	1.61^(b)^^y^		<0.001		
Mean	3.89^v^	2.91^x^	3.25^w^	1.81^y^	1.68^y^		<0.001		
*P*-values	0.56	0.028	0.020	0.030	0.056				

a–c *Within a column, means without a common superscript differ, P < 0.05; superscripts in brackets indicate a trend of a difference among means, P < 0.10*.

v–z *Within a row, means without a common superscript differ, P < 0.05*.

*SEM, the overall standard error of the mean*.

**Table 5:** Effects of the inhibition of different rumen microbial groups and of the supplementation of different oilseeds on C_4_ and C_5_ fatty acids.

**Traits/oilseeds**	**Treatment**					**SEM**	***P*****-values**		
	**Intact rumen fluid**	**–Archaea**	**–Fungi**	**–Protozoa**	**Only bacteria**		**Treatment**	**Oilseed**	**Interaction**
**Butyrate (mmol mol**^−1^**)**						2.5	< 0.001	0.98	0.46
Control	111^xy^	123^x^	101^b^^y^	63^b^^z^	64^a^^b^^z^		< 0.001		
Safflower	111^x^	120^x^	101^b^^y^	66^a^^b^^z^	65^a^^b^^z^		< 0.001		
Poppy	108^y^	119^x^	100^b^^y^	69^a^^z^	68^a^^z^		< 0.001		
Camelina	112^xy^	122^x^	104^a^^y^	64^b^^z^	62^b^^z^		< 0.001		
Mean	110^y^	121^x^	101^y^	66^z^	65^z^		< 0.001		
*P*-values	0.45	0.19	< 0.001	0.004	0.036				
**Iso-butyrate (mmol mol**^−1^**)**						0.372	< 0.001	0.003	0.64
Control	8.31^(b)^^y^	8.33^a^^y^	9.02^a^^y^	8.82^y^	14.98^(ab)^^x^		< 0.001		
Safflower	9.22^(ab)^^xy^	7.41^a^^b^^y^	7.78^a^^b^^y^	6.67^y^	12.26^(b)^^x^		0.002		
Poppy	8.36^(b)^^y^	6.47^b^^y^	6.34^b^^y^	7.48^y^	13.81^(ab)^^x^		0.003		
Camelina	9.96^(a)^^y^	8.28^a^^y^	8.78^a^^y^	8.99^y^	17.60^(a)^^x^		< 0.001		
Mean	8.96^y^	7.62^y^	7.98^y^	7.99^y^	14.66^x^		< 0.001		
*P*-values	0.035	0.011	0.008	0.30	0.077				
**Valerate (mmol mol**^−1^**)**						0.28	< 0.001	0.039	0.003
Control	11.9^b^^x^^y^	12.3^b^^x^	11.1^y^	8.0^(a)^^z^	7.4^z^		< 0.001		
Safflower	13.0^a^^x^	12.4^b^^x^	12.1^x^	7.1^(ab)^^y^	7.2^y^		< 0.001		
Poppy	12.1^b^^x^	11.9^b^^x^	12.8^x^	6.9^(b)^^y^	8.5^y^		< 0.001		
Camelina	13.1^a^^x^^y^	13.9^a^^x^	12.4^y^	8.0^(a)^^z^	7.0^z^		< 0.001		
Mean	12.5^x^	12.6^x^	12.1^x^	7.5^y^	7.5^y^		< 0.001		
*P*-values	0.002	0.008	0.19	0.037	0.18				
**Iso-valerate (mmol mol**^−1^**)**						0.522	0.004	0.007	1.00
Control	14.8^b^	17.2^a^	19.0^a^^b^	17.4^a^	14.4		0.25		
Safflower	16.5^a^^b^	16.3^a^^b^	18.4^a^^b^	15.3^a^^b^	14.9		0.79		
Poppy	14.4^b^^x^^y^	14.3^b^^x^^y^	16.5^b^^x^	13.3^b^^x^^y^	11.6^y^		0.048		
Camelina	17.1^a^	17.3^a^	19.9^a^	17.3^a^	15.3		0.39		
Mean	15.7	16.3	18.5	15.8	14.1		0.38		
*P*-values	0.007	0.034	0.034	0.010	0.13				

a, b *Within a column, means without a common superscript differ, P < 0.05; superscripts in brackets indicate a trend of a difference among means, P < 0.10*.

x–z *Within a row, means without a common superscript differ, P < 0.05*.

*SEM, the overall standard error of the mean*.

**Table 6:** Effects of the inhibition of different rumen microbial groups and of the supplementation of different oilseeds on methane and hydrogen formation.

**Traits/oilseeds**	**Treatment**					**SEM**	***P*****-values**		
	**Intact rumen fluid**	**–Archaea**	**–Fungi**	**–Protozoa**	**Only bacteria**		**Treatment**	**Oilseed**	**Interaction**
**Methane (mL g**^−1^ **dry matter)**						1.52	<0.001	0.10	0.70
Control	37.9^a^^x^	ND	21.0^a^^y^	5.7^a^^z^	ND		<0.001		
Safflower	32.3^b^^x^	ND	18.7^b^^c^^y^	5.2^a^^b^^z^	ND		<0.001		
Poppy	33.6^b^^x^	ND	20.2^a^^b^^y^	5.1^a^^b^^z^	ND		<0.001		
Camelina	34.1^b^^x^	ND	18.3^c^^y^	4.7^b^^z^	ND		<0.001		
Mean	34.5^x^	ND	19.5^y^	5.2^z^	ND		<0.001		
*P*-values	<0.001	–	0.002	0.043	–				
**Methane (mL g**^−1^ **organic matter digested)**						2.31	<0.001	0.28	0.94
Control	58.5^a^^x^	ND	36.8^a^^y^	11.7^z^	ND		<0.001		
Safflower	53.0^b^^x^	ND	33.9^b^^c^^y^	11.0^z^	ND		<0.001		
Poppy	54.1^b^^x^	ND	35.3^a^^b^^y^	10.6^z^	ND		<0.001		
Camelina	54.4^b^^x^	ND	32.4^c^^y^	10.3^z^	ND		<0.001		
Mean	55.0^x^	ND	34.6^y^	10.9^z^	ND		<0.001		
*P*-values	<0.001	–	<0.001	0.17	–				
**Methane (mmol mol**^−1^ **total short-chain fatty acids)**						6.0	<0.001	0.99	0.99
Control	129^x^	ND	71^y^	20^z^	ND		<0.001		
Safflower	123^x^	ND	72^y^	21^z^	ND		<0.001		
Poppy	125^x^	ND	75^y^	19^z^	ND		<0.001		
Camelina	128^x^	ND	68^y^	18^z^	ND		<0.001		
Mean	126^x^	ND	71^y^	19^z^	ND		<0.001		
*P*-values	0.63	–	0.35	0.60	–				
**Gaseous hydrogen (gH2; mL g**^−1^ **dry matter)**						0.997	<0.001	0.13	0.25
Control	0.04^y^	30.20^a^^x^	0.61^y^	0.39^y^	2.04^a^^y^		<0.001		
Safflower	0.03^y^	26.63^b^^x^	0.22^y^	0.22^y^	1.49^b^^y^		<0.001		
Poppy	0.03^y^	26.35^b^^x^	0.41^y^	0.32^y^	1.60^b^^y^		<0.001		
Camelina	0.05^y^	26.33^b^^x^	0.86^y^	0.25^y^	1.50^b^^y^		<0.001		
Mean	0.04^y^	27.38^x^	0.52^y^	0.29^y^	1.66^y^		<0.001		
*P*-values	0.34	0.002	0.27	0.40	<0.001				

a–c *Within a column, means without a common superscript differ, P < 0.05*.

x–z *Within a row, means without a common superscript differ, P < 0.05*.

*SEM, overall standard error of the mean. ND, not detected*.

**Table 7:** Effects of the inhibition of different rumen microbial groups and of the supplementation of different oilseeds on calculated hydrogen balance and dissolved hydrogen.

**Traits/oilseeds**	**Treatment**					**SEM**	***P*****-values**		
	**Intact rumen fluid**	**–Archaea**	**–Fungi**	**–Protozoa**	**Only bacteria**		**Treatment**	**Oilseed**	**Interaction**
**H**_2_ **Generated (mmol)**						0.083	<0.001	<0.001	0.89
Control	4.87^x^	4.57^b^^y^	4.64^b^^y^	3.70^b^^z^	3.57^(b)^^z^		<0.001		
Safflower	5.01^x^	4.75^a^^y^	4.81^a^^b^^y^	3.79^a^^b^^z^	3.70^(ab)^^z^		<0.001		
Poppy	5.14^x^	4.64^a^^b^^x^	4.77^a^^b^^x^	3.74^b^^y^	3.62^(ab)^^y^		<0.001		
Camelina	5.22^x^	4.80^a^^y^	4.92^a^^y^	3.87^a^^z^	3.71^(a)^^z^		<0.001		
Mean	5.06^x^	4.69^y^	4.78^y^	3.77^z^	3.65^z^		<0.001		
*P*-values	0.25	0.005	0.007	<0.001	0.054				
**H**_2_ **Utilized (mmol)**						0.050	<0.001	<0.001	0.99
Control	2.60^b^^x^	1.55^c^^z^	2.12^b^^y^	1.74^b^^z^	1.61^b^^z^		<0.001		
Safflower	2.66^b^^x^	1.61^a^^b^^z^	2.21^a^^b^^y^	1.83^a^^z^	1.67^a^^b^^z^		<0.001		
Poppy	2.67^b^^x^	1.58^b^^c^^z^	2.23^a^^y^	1.81^a^^z^	1.66^b^^z^		<0.001		
Camelina	2.83^a^^x^	1.66^a^^z^	2.25^a^^y^	1.87^a^^z^	1.74^a^^z^		<0.001		
Mean	2.69^x^	1.60^z^	2.20^y^	1.81^z^	1.67^z^		<0.001		
*P*-values	<0.001	0.001	0.007	0.001	<0.001				
**H**_2_ **Recovered (%)**						0.69	<0.001	0.55	0.99
Control	53.7^x^	33.9^b^^z^	45.5^y^	47.0^b^^y^	45.1^b^^y^		<0.001		
Safflower	53.4^x^	34.0^b^^z^	45.5^y^	48.4^a^^y^	45.5^a^^b^^y^		<0.001		
Poppy	53.3^x^	34.1^b^^z^	46.4^y^	48.5^a^^x^^y^	46.0^a^^b^^y^		<0.001		
Camelina	54.5^x^	34.7^a^^z^	45.3^y^	48.3^a^^y^	47.1^a^^y^		<0.001		
Mean	53.7^x^	34.2^z^	45.7^y^	48.1^y^	45.9^y^		<0.001		
*P*-values	0.75	0.009	0.57	0.007	0.039				
**Dissolved H**_2_ **(**μ**M)**						2.867	<0.001	0.49	0.95
Control	0.11^z^	82.44^a^^x^	2.06^y^^z^	1.91^y^^z^	10.27^a^^y^		<0.001		
Safflower	0.09^z^	80.65^a^^b^^x^	0.93^z^	1.19^y^^z^	8.34^b^^y^		<0.001		
Poppy	0.08^z^	77.39^b^^x^	1.45^z^	1.66^z^	8.49^b^^y^		<0.001		
Camelina	0.15^z^	80.56^a^^b^^x^	3.74^y^^z^	1.35^y^^z^	8.05^b^^y^		<0.001		
Mean	0.10^z^	80.26^x^	2.04^y^^z^	1.53^z^	8.79^y^		<0.001		
*P*-values	0.36	0.039	0.17	0.55	<0.001				

a–c *Within a column, means without a common superscript differ, P < 0.05; superscripts in brackets indicate a trend of a difference among means, P < 0.10*.

x–z *Within a row, means without a common superscript differ, P < 0.05*.

*SEM, overall standard error of the mean*.

### General effects of the microbial groups on fermentation and methane emission

The mean pH of the incubation fluid from treatments –A and –F was lower (*P* < 0.05) and that of the treatments –P and –AFP was higher (*P* < 0.05) compared to treatment I (Table 3). The ammonia concentrations decreased (*P* < 0.05) by the application of each of the inhibitors compared to I. The OM digested and IVOMD were smaller (*P* < 0.05) in –F, –P, and –AFP than in I, whereas –A had no effect. Inhibitors decreased (*P* < 0.05) total SCFA concentration in –A, –P, and –AFP compared to I, with the lowest values found with –P and –AFP (Table 4). All inhibitors increased (*P* < 0.05) propionate proportion and decreased (*P* < 0.05) acetate proportion as well as acetate-to-propionate ratio. Compared to I, the proportions of butyrate and valerate were smaller (*P* < 0.05) in –P and –AFP, but not in –A and –F (Table 5). The proportion of iso-butyrate was greater (*P* < 0.05) in –AFP compared to I. There were no inhibitor effects on iso-valerate proportion. Inhibition of the archaea (–A and –AFP) completely prevented CH_4_ production (Table 6). Also –F and especially –P diminished (*P* < 0.05) CH_4_ production expressed either per gram of DM, per gram of OM or per unit of SCFA. In –A the formation of gH_2_ was higher (*P* < 0.05) than in all other treatments. The calculated amounts of total H_2_ generated, H_2_ utilized and H_2_ recovered were highest (*P* < 0.05) in I (Table 7). The calculated H_2_ recovery was lowest (*P* < 0.05) in –A and intermediate (*P* < 0.05) in –F, –P, and –AFP. The calculated amount of H_2_ generated was lowest (*P* < 0.05) in –P and –AFP, and the calculated amount of H_2_ utilized was lowest (*P* < 0.05) in –A, –P, and –AFP. The estimated amount of dissolved H_2_ was highest (*P* < 0.05) in –A, followed by –AFP and then by the other treatments –F, –P and I.

### Effect of oilseeds in the different microbial treatments

There were interactions (*P* < 0.05) between oilseed treatment and microbial treatment for pH, ammonia concentration and valerate proportion of total SCFA. In the case of pH, the oilseeds had a lowering (*P* < 0.05) effect in –F, –P, and –AFP, an effect not observed in I and –A (Table 3). For changes in ammonia concentration, the effects of the oilseeds were mostly smaller in extent when inhibitors were used. The same is true for valerate proportion where effects of oilseed were only found with –A (*P* < 0.05) and –P (*P* < 0.10), but not in –F and –AFP (Table 5). The CH_4_ mitigating effect of some oilseeds disappeared with the inhibitor treatments –F and –P. In –F, the poppy seed supplementation did not reduce CH_4_ production. In –P, only camelina seeds caused a decrease (*P* < 0.05) in CH_4_ production (Table 6). All oilseeds reduced (*P* < 0.05) gH_2_ production compared to the control diet in treatments –A and –AFP, even though no significant interaction was found. In all of the inhibitor treatments, some of the oilseeds enhanced (*P* < 0.05) the calculated amounts of H_2_ generated and H_2_ utilized as well as H_2_ recovery (Table 7). All oilseeds reduced (*P* < 0.05) the estimated amount of dissolved H_2_ compared to the unsupplemented control diet in treatment –AFP. In –A, only poppy seeds caused a decrease (*P* < 0.05) in the estimated dissolved H_2_.

## Discussion

### Success of elimination of distinct microbial groups and selectivity of the inhibitors

We selected inhibitors found efficient individually in a number of other investigations. The compound BES is a potent CH_4_ inhibitor, but the actual extent of suppression of CH_4_ emission was found to be variable. Martin and Macy (1985) described a decrease in methanogenesis by 76% after 2 h of incubating rumen fluid *in vitro* with 0.030 mmol BES L^−1^. Dong et al. (1999) observed that 0.072 mmol BES L^−1^ reduced CH_4_ emission by 51% in Rumen Simulation Technique. An amount of 20 mmol BES L^−1^ completely inhibited *in vitro* CH_4_ formation after 2 h in the study of Poulsen et al. (2013), similar to results in the present study. Cycloheximide has been suggested (Akin and Benner, 1988; Dehority and Tirabasso, 2000) to inhibit the fungal growth in rumen fluid when used at the concentration applied in the present experiment. For the removal of the protozoa (defaunation), Synperonic was the most effective agent in the study of Dohme et al. (1999). The agent was highly efficient in the present study as well.

In our preliminary experiment, the proportion of CH_4_ in total gas produced decreased with all inhibitors compared to the intact rumen fluid. In compensation to this, gH_2_ clearly increased in the –A treatment, which was already observed at the first measurement time point (1 h of incubation). The gas concentrations approached a plateau afterwards. This indicates that the activities of targeted microorganisms were permanently impaired or totally inhibited. The inhibitor BES used in the treatments –A and –AFP is a structural analog of coenzyme M (Co-M) and directly competes with Co-M for the methyl group (Guo et al., 2008). By this way, BES inhibits the activity of the methyl-Co-M reductase in archaea and thus the CH_4_ production (Waghmode et al., 2015). In the present study, no CH_4_ emission was detected in the treatments with BES and correspondingly the abundance of *mcrA* gene coding for alpha subunit of methyl-Co-M reductase enzyme was reduced by up to 96% (–A) and 98% (–AFP). At the same time there was still total archaeal RNA present at a level of 33% of control (intact rumen fluid). Agarwal et al. (2008) observed a reduction of 98% in CH_4_ emission with BES treatment *in vitro* while archaeal DNA was found at a level of 14% of control. That means CH_4_ formation can be at zero level at a time when archaeal RNA (and DNA) is still present. The microbial data from the treatment with the combination of all three inhibitors showed that the three agents did not interact.

The inhibitor technique used in the present study has its limitations concerning the association of the effects to groups of microorganisms by being inhibitory to more than the target group, by compensatory actions of other microorganisms, by affecting synergistic microorganisms or a combination thereof. The interactions in the rumen microbial ecosystem are very complex because of the diversity and various synergistic and antagonistic actions (Orpin and Joblin, 1997). In the present study, the inhibition of the protozoa (–P and –AFP) resulted in the expected compensatory increase in the activity of the bacteria (measured as relative quantity of RNA abundance). The predation of bacteria by protozoa is known for long (Morgavi et al., 2010). Defaunation prevents the associated loss of bacterial protein, thus improves the efficiency of total microbial protein synthesis. Synperonic used for defaunation led to a partial inhibition of the archaea in the present study. This can be explained by the share of up to 25% of the archaea, which are endo- and ecto-symbiotically associated with the protozoa (Newbold et al., 1995; Morgavi et al., 2010). A recent meta-analysis by Newbold et al. (2015) showed that defaunation also impairs fibrolytic microorganisms such as fungi, *R. albus* and *R. flavefaciens*, which is consistent with the results of the present study. The numerical increase in total bacterial RNA abundance when inhibiting fungi might be due to the removal of competition for nutrients. Gordon and Phillips (1993) examined the combined addition of tetronasin and cycloheximide to inhibit the ruminal fungi in sheep and found that total viable bacteria, and cellulolytic bacteria numerically increased and protozoa numerically decreased, which is in agreement with the results of the present study. The increase in total bacterial RNA abundance when inhibiting the methanogens could be a compensation for the assumed impaired efficiency of ruminal fermentation caused by increasing levels of H_2_. The current results from treatment –A with the (numerical) decrease in total fungal RNA and the (numerical) increase in *F. succinogenes* RNA abundance are in agreement with the findings of Guo et al. (2007) based on the DNA abundance of these (groups of) microorganisms.

### Effect of the oilseeds

In the intact rumen fluid, the addition of the seeds to the basal diet affected ammonia concentration and OM digestion likely resulting from the shift among fermentable nutrients by the inhibitory effects of the lipids. Especially, the effects of CH_4_ mitigation found in a previous *in vitro* experiment carried out with the same batches of these three uncommon seeds (Wang et al., 2017) were confirmed. All seeds decreased CH_4_ production by 10–15% when related to grams of DM supplied and by 7–9% when related to grams of OM digested. There were no significant differences among the oilseeds in this respect. Differences in effect among the oilseeds were observed for H_2_ utilization, which was higher compared to the control with the addition of camelina, but not safflower and poppy seeds. The addition of camelina was also most effective in changing the proportions of the minor SCFA. Based on the review by Beauchemin et al. (2008), the effectiveness of mitigating CH_4_ by supplementing lipids depends on many factors apart from lipid level, among them the fatty acid composition, even though this was not causative in each experiment. As lipid level was kept constant in the present study, the differences observed among the seeds are most likely related to the respective fatty acid profile. Camelina seeds were rich in a fatty acid with three double bonds whereas poppy seeds and especially safflower seeds were rich in a fatty acid with only two double bonds.

### Effects of the microbial groups

Archaea are the sole producers of CH_4_ in the rumen. Their inhibition with BES led to a drastic increase in the release of gH_2_ and estimated dissolved H_2_, as the hydrogen was no longer removed by the archaea. This coincides with findings of Wang et al. (2016b). Such an accumulation of gH_2_ and dissolved H_2_ is accompanied by changes in the H_2_ balance and fermentation pattern (Janssen, 2010; Wang et al., 2016b; Guyader et al., 2017). Correspondingly, in the present study the amounts of H_2_ utilized and recovered were decreased as the result of the CH_4_ depletion, even though the partial shift in the rumen fluid SCFA profile from acetate to propionate utilized extra H_2_. This was consistent with the meta-analysis by Ungerfeld (2015). It has been assumed that the increasing pressure of H_2_ could decrease the efficiency of ruminal fermentation by impairing the function of microbial enzymes (Morgavi et al., 2010). However, Dittmann et al. (2016) reported that the administration of the CH_4_ inhibitor bromochloromethane to cows increased the digestibility of crude protein and fiber at substantially decreased CH_4_ yield. No data on gH_2_ was available from that experiment. In the present study, selectively inhibiting the archaea had no effect on IVOMD. The trend toward an increase in total bacteria RNA abundance (1.7-fold) observed in this treatment might explain why an efficient OM degradation could be maintained. Also Guo et al. (2007) found an increased *F. succinogenes* population and a decreased fungal population with BES treatment and no effect on fiber digestibility. Thus, it might be an alternative way by promoting non-H_2_-producing microorganisms such as *F. succinogenes* to decrease CH_4_ emissions in the rumen without impairing fiber digestibility as suggested by Morgavi et al. (2010).

Fungi inhabiting the rumen synthesize and secrete a wide range of highly active enzymes for the degradation of cell wall carbohydrates (Orpin and Joblin, 1997). Often they perform the primary colonization of the plant cell wall, thus making it accessible to other microorganisms (Theodorou and France, 2005). In the present study, IVOMD and total SCFA production decreased by inhibiting the fungi, illustrating their importance in this respect. Fungi also possess a high proteolytic activity (Wallace and Joblin, 1985; Nolan and Dobos, 2005), which explains the decline in ammonia concentration without fungal activity. In the present study, the decline in CH_4_ after eliminating the fungi was associated with a reduced H_2_ generation and a shift in the fermentation pattern from acetate to propionate, which competes with methanogenesis for H_2_ utilization.

The inhibition of the protozoa led to a clear reduction in CH_4_ production in the present study, but the compensatory increase in gH_2_ and estimated dissolved H_2_ was quite small. Protozoa not only supply archaea with H_2_ but also protect them from oxygen which is toxic to archaea (Morgavi et al., 2010). The inhibition of the protozoa decreased also ammonia concentration, IVOMD and total SCFA formation and the propionate proportion increased at the expense of the other SCFA. Such effects of protozoa inhibition are similar to those reported using complete defaunation (e.g., Dohme et al., 1999) and to the description in a number of review articles (e.g., Newbold et al., 2015; Tapio et al., 2017).

The treatment of the rumen fluid with a combination of antimethanogenic, antifungal and antiprotozoal agents, leaving almost only bacteria RNA in the medium, resulted in zero CH_4_ production in the present study. However, even though gH_2_ generation and the estimated amount of dissolved H_2_ was increased by this treatment (–AFP), the increase was by far lower compared to that occurring with the inhibition of archaea only (–A). This indicates that the inhibition of the fungi and the protozoa and changes in the composition of the bacterial group reduced excessive H_2_ generation. This is also obvious from the changes in H_2_ balance. The concomitant decline in IVOMD, total SCFA and ammonia concentration in –AFP indicates that the interplay of the microbial groups is important for an effective ruminal nutrient degradation. This especially concerns fiber digestion as can be seen in the change of fermentation pattern from acetate to propionate formation in –AFP.

### Role of the microbial groups for the methane-suppressing effect of the oilseeds

Supplementation of oilseeds in the absence of archaeal activity (–A) led to a decrease in the gH_2_ generated (all oilseeds) and dissolved H_2_ (poppy seeds) and to an increase in the amounts of H_2_ utilized (safflower and camelina seeds) and recovered (camelina seeds). This might be the result of PUFA biohydrogenation, even when considering that the proportion of H_2_ captured by this way was estimated to account for only 1–2% (Czerkawski and Clapperton, 1984). This phenomenon might be restricted to situations where the CH_4_ pathway was blocked, because no such oilseed effects were observed with intact rumen fluid. Therefore, in the presence of the archaea the majority of metabolic hydrogen seems to be utilized to form CH_4_.

The effects of oilseeds and those of the removal of the fungi seemed to be widely independent and synergistic, resulting in a final CH_4_ suppression per unit of digestible OM of up to 45% in comparison to intact rumen fluid incubated with the basal diet as sole substrate. This contradicts the suggestion that PUFA, by inhibiting the growth of the cellulolytic ruminal fungi (Maia et al., 2007; Zhang et al., 2008), exert indirect effects via decreasing the H_2_ production. In the present study, some differences between the oilseeds in this context were observed. With camelina seeds, the extent of suppression of CH_4_ per unit of digestible OM was by tendency higher in the absence of the fungal activity than with intact rumen fluid (−12 vs. −7%, *P* = 0.06). No difference was found with safflower seed and with poppy seeds the opposite occurred (−4% with –F vs. −8% with I, *P* = 0.03). This indicates that α-linolenic acid with three double bonds, prevalent in camelina, reduces CH_4_ emission mainly by its toxicity against bacteria or archaea. While linoleic acid with two double bonds, prevalent in poppy (but also in safflower) seeds, could have exerted an effect through the toxicity against the fungi. This is in agreement with the report on the toxicity of linoleic acid against fungi by Maia et al. (2007). An interaction was found between oilseed and anti-fungal treatments in ammonia concentration in a way that the decrease caused by the anti-fungal treatment, otherwise obvious, was absent with poppy seeds. This suggests that in the case of poppy seed addition, the fungi were not responsible for part of the extra ammonia formation.

The levels of suppression of CH_4_ emission per unit of DM and OM digested caused by the oilseed supplementation were clearly smaller in protozoa-inhibited than in intact rumen fluid with safflower or poppy seeds. Whereas with camelina seeds the absence of protozoa enhanced the extent of methane suppression per g DM (18 vs. 10% with I, *P* = 0.08). This confirms the important role of the protozoa for the CH_4_ suppressing effect of the lipid. The susceptibility of the protozoa against various type of lipids (at similar concentrations as used in the present study) is well known and may even approach complete defaunation (Dohme et al., 1999, 2001). Still, Dohme et al. (1999) showed that the effects of supplementing lipids (coconut oil) and chemical (synperonic NP9) defaunation, both effective in CH_4_ suppression (48 and 52%, respectively) can be additive and they resulted in a 90% CH_4_ suppression in that study. The increase in ammonia concentration with the oilseeds was less pronounced without than with protozoal activity. Protozoa are known to produce high amounts of ammonia not only from feed protein but also from degrading bacterial protein (Newbold et al., 2015).

Manipulation of the rumen fluid to prevail bacteria only, resulted in a lower production of gH_2_ and estimated dissolved H_2_ in the presence of the oilseeds. This was not observed in the intact rumen fluid likely due to the generally minute amounts of gH_2_ and estimated dissolved H_2_ released. This observation shows that the bacteria are active in utilizing excessive H_2_ in order to biohydrogenate PUFA from the oilseeds. Among the oilseeds, camelina seeds took a special position. In the bacteria-only treatment but not in the intact rumen fluid, the effect of camelina seeds on calculated H_2_ production and utilization as well as proportions of some SCFA differed from those of safflower and poppy seeds. The oilseeds caused changes in ammonia concentration, which were smaller when only the bacteria were present, indicating that bacteria were least responsible from the microbial groups in this respect.

## Conclusion

In the present study, effects of individual microbial groups (archaea, fungi, protozoa, and bacteria) and individual oilseeds were numerous and complex. The interactions between oilseeds and microbial groups were important for the antimethanogenic effect of oilseeds. The antimethanogenic effect of camelina seeds was directed specifically against bacteria and archaea and was weakened by the presence of fungi and protozoa. The CH_4_ lowering effect caused by safflower and poppy seeds occurred predominantly through interactions with protozoa or protozoa-associated archaea, and the presence of the fungi enhanced the effect in case of poppy seeds. These findings help to understand how lipids of different composition are mitigating CH_4_ emission, dependent on the presence of a microbial group. Thereby, these findings facilitate the strategic application of lipids in ruminant nutrition. Nevertheless, these *in vitro* results need to be interpreted with caution when extrapolated to *in vivo* conditions and should be confirmed in live animals.

## Author contributions

SW, MK, KG, and AS designed this experiment. SU contributed the microbial analysis instruments and UB provided the access to the rumen-cannulated Brown Swiss cow. SW conducted the experiment, performed the statistics and drafted the manuscript. MK, KG, SU, UB, and AS contributed in revising critically the manuscript. All authors read and approved the final manuscript.

### Conflict of interest statement

The authors declare that the research was conducted in the absence of any commercial or financial relationships that could be construed as a potential conflict of interest.
